# Gangrenous Degeneration of the Cheek and Gums, with Necrosis and Exfoliation of the Alveolar Processes and Maxillary Bone

**Published:** 1857-01

**Authors:** J. Richardson

**Affiliations:** Cincinnati.


### ARTICLE XXI.

### *Gangrenous Degeneration of the Cheek and Gums, with Necrosis and Exfoliation of the Alveolar Processes and Maxillary Bone.* By J. Richardson, D. D. S., M. D., Cincinnati.

Case.—Child (female) æt. four years, of an asthmatic predisposition. Has had frequent attacks of *congestive bronchitis*, which under the care of the family physician, have always yielded readily to judicious treatment. Upon the accession of a similar attack in January last, however, the regular medical attendant being absent, its management was intrusted to an irregular practitioner. Unpromising symptoms rapidly supervening, the usual family adviser was called to the case on the fifth day after the commencement of the disease, and found the little patient suffering severely under all the more urgent symptoms usually accompanying that form of disease. Improper, as well as inefficient treatment, had confirmed and rendered less tractable the morbid conditions designed to be alleviated. The malady was characterized by a feeble, frequent pulse, obstructed capillary circulation throughout, an imperfect action of all the functions of secretion, embarrassed respiration, and great enfeeblement of the vital powers. These untoward symptoms gradually increased in severity up to the eighth or ninth day from the attack, at which time the child was threatened with speedy dissolution from impending asphyxia. At this critical juncture, and as a last resort, four leeches were applied to the chest. Happily, this direct depletion of the parts was followed by an obvious mitigation of the more urgent symptoms. Convalescence, however, though dating from this time, was tardy and uncertain, being obstructed in part, doubtless, by serious infiltration into the submucous cellular structure of the bronchia, but chiefly by an accidental complication to be mentioned directly. We would premise a description of the latter by a brief allusion to a portion of the treatment before leeching, as pertinent to some considerations that will be alluded to hereafter. *Mercurials*, with such other remedies as the case seemed to require, were given in conjunction with nauseants, with a view to their alterative action upon the liver, in all about four grains. No perceptible action upon that organ was obtained, the remedy was discontinued, and never afterwards renewed. Counter-irritation over the chest by the use of croton oil was likewise attempted, but instead of healthy pustulations it provoked but an ill-conditioned sore, with a strong tendency to sloughing, a result attributable to a marked deterioration of the healthy constitution of the blood, and a consequent weakening of the vital resistance in the tissues.

Whilst a gradual improvement was being effected in the general condition of the patient, a new aspect of diseased action manifested itself, which, in its subsequent development, became a source of considerable anxiety and alarm. On the third or fourth day after the leeching, a small ulcerated spot, of a dirty yellowish hue, about the size of half a pea, made its appearance on the labial portion of the gums corresponding with the left second superior molar tooth, and lying closely upon the outlet of the salivary duct. The first indication of its presence was a peculiar glazed appearance of the cheek externally, accompanied with some tumefaction. In a short time the disorganizing process within had involved a considerable portion of the cheek; the parts as they became progressively implicated, presenting all the physical characteristics of complete sphacelus prior to disintegration, but without any apparent tendency to sloughing. As it dipped deeper into the substance of the cheek, its integuments externally presented varying conditions. From its original smooth, shining aspect, it assumed next a diffused blush or redness, then a more dusky appearance, and finally, deepening in color, a circumscribed portion began to assume a decided livid hue, surrounded by a grayish areola. Within, the gangrenous portions extended from the posterior molar to within about a quarter of an inch of the angle of the mouth, in width about three-quarters of an inch, and in depth nearly the entire thickness of the cheek. At this stage, the indications furnished ground for the most painful apprehensions. *Perforation of the cheek*, an issue fraught only with evil, and sufficiently lamentable in its mildest aspect, seemed inevitable. Its termination, however, contrary to every reasonable expectation, was otherwise. The integuments remained *intact*, and the little patient was saved from the consequences of mutilation. Under a constitutional treatment, dating back for several days—a treatment at once prompt, vigorous, and unwavering, addressed to the impaired nutritive functions, the local malady, though not perceptibly checked in its progress for several days, was, we have good reason to infer, by these means, ultimately held in abeyance, at a most critical time, for some forty-eight hours, during which the destructive process seemed neither to advance nor retrograde. At the conclusion of that time, amendment was perceptible, the discoloration of the cheek began gradually to give way, the slough to detach itself slowly at the edges, reparation by granulation in due time commenced, and the upbuilding of the wasted structure was finally carried on to a successful issue.

The disease, in its invasion of the gums, was in part concurrent with that of the cheek, but was more tardy and protracted, continuing its encroachments in that direction for a time after a favorable change in the latter. It ultimately involved all that portion investing and surrounding the molar teeth, invading successively the maxillary and alveolo-dental periosteum, the alveolar processes, and a portion of the maxilla proper. Its further progress was however, in a short time, arrested, exfoliation of the necrosed portions effected, and a healthy local action promoted by the use of appropriate topical appliances.

The affection throughout was progressively circumscribed, and seemed to be propagated by mere *extension* from the original seat. The gums, tongue, fauces, glands, mucous membrane, etc., outside of the immediate action of the destructive process, preserved an entirely healthy aspect. This fact, in view of the liability of confounding this affection with other morbid conditions of the mouth, is invested with some importance as a distinctive feature of certain phenomena on which a correct diagnosis may be founded.

We had an opportunity a few days since of examining this little girl's mouth. There is no apparent contraction, but a marked *scooping-out* of the jaw in the space affected. As there will be no reproduction of the processes, this condition, of course, will be permanent. The undeveloped crowns of the permanent bicuspids, which immediately presented on the removal of the superincumbent mass, were so far implicated in the disease that they were, I am informed, spontaneously cast within a few weeks. Considerable constriction exists about the cheek, owing to adhesive inflammation having to some extent rendered reflected portions of the mucous membrane adherent. There is also some want of concerted action in the movements of the affected side, referable, probably, to imperfect or irregular supply of nervous influence to the parts. To what extent the imperfectly developed permanent cuspid and anterior molar have been affected by the disease, we have no means of determining. If erupted at all, their organization will almost certainly be defective, either in whole or at some particular points, from interrupted or perverted nutrition at the time.

Several points of practical interest present themselves in connection with the *cause*, *pathology*, *diagnosis*, and *treatment* of this and similar cases. Although such may not generally come within the range of dental practice, yet it not unfrequently happens that members of the profession are called upon to pass judgment upon the good faith or competency of others, whose interests or reputation may be seriously affected by the opinion given. Our own personal credit, therefore, stands pledged to a guarded and intelligent response, and the fair fame of our profession, which purports to be a *scientific* and an *educated* one, our obligations to the patient or friends, and the best interests of the medical attendant, as well as the impulse of common honesty, demand that our judgments shall be fortified by such medical attainments as will preclude, as far as may be, the possibility of mistake.

The foregoing case will be readily recognized as one in which popular impeachment but too often follows closely upon popular error or prejudice. The physician, innocent of the wrong charged, is made to smart under the censorious criminations of ignorance or malice, by which public confidence in his skill, honesty, or discretion is weakened. We refer to an *educated* proclivity in the common mind to ascribe almost every form of diseased action about the mouth, characterized by any considerable destruction of its tissues, to the *excess of mercurial salivation.* If *calomel* (a remedy dangerous only in the hands of the unskillful) is given, no matter when or how minutely or how cautiously, a complication like the foregoing becomes at once a topic for injurious criticism, or cause for the infliction of unmerited odium, from which the implicated party has no sufficient appeal. It is, therefore, important that we should be able to discriminate between the local phenomena of mercurial ptyalism and other and far different conditions of morbid action. We owe it to ourselves, to the public, to our medical brethren, and to a remedy invaluable and indispensable as a therapeutic agent.

*Cause and Pathology.*—The local disease under consideration was unquestionably one of *debility*, and must not be confounded with any particular vice of the system, or with any diathesis

*specifically* prone to this form of disease, nor, especially, with the action of mercurials. An inquiry into the probable agencies contributing to this affection will elucidate the caution. There were two concurrent conditions prevailing in this case—one *predisposing* and having a systemic origin; the other local in its immediate action and constituting the *exciting* cause. The predisposition consisted essentially in impaired nutrition and in great enfeeblement of the vital forces, induced by a protracted and exhausting disease. Constitutions greatly weakened by any causes operating to impair the integrity of the organism are strongly prone to textural disorganization. This tendency prevails especially among children subjected habitually to the unwholesome influences of bad food, impure air, personal filthiness, etc., though not necessarily dependent upon these circumstances for its development, as in the present instance.

The *exciting* cause consisted in a *vitiation* of the *salivary secretions*. In *health*, the laws of waste and reproduction operate harmoniously. All the actions concerned in assimilation and elimination are mutual and compensating; elaborated material for cell-growth is duly appropriated, and the effete products silently removed through their appropriate channels—all the functions, in fine, are performed in obedience to laws having a physiological determination. In *disease*, however, especially in the phlegmasia and in febrile affections, the fluids of the body become surcharged with noxious accumulations, the appointed scavengers of the system are thereby over-taxed, and organs which, in a state of health, are employed in the elaboration of healthy secretions, are burdened with vicarious functions for the depuration of unwholesome material.

We find a solution of the causes in this case, therefore, in the conditions mentioned, namely, impaired vital resistance as a sequence of perverted nutrition or ultimate molecular degeneration and constitutional debility, co-operating with a fluid (salivary) naturally bland, but rendered depraved and acrimonious by the admixture of degenerate effete products. This view receives confirmation in the circumstances surrounding the local phenomena, as well as in the results of treatment. Topical applications which, in diseases of the mouth, uncomplicated with vital prostration, are *curative* in their action, instead of controlling or mitigating the symptoms, as primary appliances, in this case, tended rather to aggravate and intensify the disease. No improvement was observed until recuperative means, addressed to the constitutional powers, were employed. We have already adverted to the manner of this action. Again, its locality and progress are suggestive. The ulcerated spot appeared first contiguous to the outlet of the parotid or Stenonian duct. The salivary secretions being partially confined in this situation by valvular folds of the mucous membrane, acted persistently and concentratively upon parts that had not the power successfully to repel their encroachments. Its subsequent extension was due to a continuance of this cause, conjointly with the erosive exhalation from, and inflammatory excitation of, the diseased mass.

*Diagnosis.*—The distinction between this affection and that phagedenic or gangrenous disease of the mouth known as cancrum oris, is not, by any means, well marked. While the latter is, perhaps *constantly* associated with a depraved or cachectic habit arising from constitutional taint, or from the want of proper food, pure air, want of cleanliness, &c., the former is but a modification or milder expression of the same disease, manifesting itself independently of these influences. They have nearly the same local phenomena in common; have both primarily a constitutional origin, and are alike diseases of debility. The distinction, if any, is not important, since the indications of treatment are the same.

From dental irritation, mechanical injuries, venereal complaints, small pox, &c., it is readily distinguishable.

Between the disease in question and the local phenomena of mercurial salivation, there are some unimportant points of correspondence, but the two are, nevertheless, widely dissimilar. Mercurial remedies, by virtue of their specific action, find expression in certain determinate local sequellæ, which may be said to be pathognomic of the disease. Among these we find a turgidity or puffiness of the gums, inflammation and thickening of the alveolo-dental periosteum, with consequent loosening of the teeth, inflammation and swelling of the salivary glands, with profuse discharges therefrom; a peculiar fetor of the breath; inflammation of the mucous membrane of the cheeks and fauces; inflammation and swelling of the tongue and cheeks, with cellular infiltration, sloughing, necrosis, &c. It will be remembered that in severe cases these symptoms are constant, general and diffused, attacking almost simultaneously the various organs implicated. In cancrum oris and its modifications, on the contrary, the "ulcération, or gangrene, is *circumscribed;* is generally confined to one side; commences usually in the cheek, and only affects that part of the gums which is in close contiguity, the tongue being untouched.*" A reference to these points of difference, which are sufficiently distinctive, will preclude, in most cases, the possibility of a false diagnosis.

*Treatment.*—The amenability of this affection to proper treatment, even under the most unpromising circumstances, was, we think, demonstrated in the favorable termination of this case. Prof. Harris, in his valuable treatise on the "*Principles and Practice of Dental Surgery,*" has the following in reference to the treatment in these cases:

"The local treatment should consist of acidulated and astringent gargles, and a solution of the chloride of lime, or soda. The ulcerated parts should be occasionally touched with a strong solution of the nitrate of silver; and Delabarre says, he has, in some cases, derived great advantage from touching them with the actual cautery. As soon as the alveolar processes exfoliate, they should be removed. After this takes place, a cure with suitable constitutional treatment, is generally speedily effected."

We make this quotation for the purpose of directing attention to a singular inconsistency into which the learned author has, singularly enough, fallen. In discussing the causes of this disease he distinctly affirms its constitutional origin. He says: "The disease seems to be the result of general debility, or defective nutrition and a cachectic habit of body." Again, "That the disease is determined by general enfeeble-

* Druitt's Surgery.

ment of the functions of the body, there is, we think, little doubt." And again, "While, therefore, the character of the affection is determined by some peculiar constitutional tendency and general enfeeblement of the vital powers of the body, it is not unlikely that local irritation is the immediate cause of its development."

Now, it is a little singular, that immediately following the enunciation of these views, which are undoubtedly correct, we should be instructed to pin our faith upon local remedies for the subduction of a mere symptom, or sequence, brought into active development by an accidental circumstance, whilst an antecedent condition of constitutional origin, and of which the former is but an outgrowth, or manifestation, is permitted to remain operative and uncontrolled. The treatment for the *cure* of the local trouble is made to consist, in the first instance, of remedies applied directly to the diseased surface, without reference to a concurrent constitutional state, furnishing continually the very conditions of its existence and perpetuation. This, if not clearly expressed by the author, is plainly inferential, for, after stating the local treatment with some particularity, as applicable to these cases, he remarks, as before quoted: "*After this* (exfoliation) *takes place*, a cure, *with suitable constitutional treatment*, is generally speedily effected." Now, it is plain, that if remedies addressed to the *primary* and *efficient cause of the disease* are deferred until *exfoliation* is effected, we may then rest upon our arms, self-satisfied with the agreeable (?) consciousness that *nature* has effected what the resources of art might have accomplished at a much earlier day, if properly directed, and that a cure is being effected and no thanks to "constitutional treatment," "*after exfoliation.*"

We have presented this somewhat extended review of the author's remarks on the treatment of these cases, in the full belief that they inculcate an important error. We believe that a practical fulfillment of the suggestion indicated by them, at the bedside, would work, in many instances, irreparable mischief. No reliance is certainly to be placed upon local applications, beyond what may be accomplished by keeping the mouth cleansed of acid discharges and the fetor moderated by appropriate washes. The reason is apparent, when considered with reference to such remedies sufficiently potent to be *curative* in their action. We have seen that the immediate cause leading directly to the disorganization of the parts, in the case before us, is referred to an inflammatory excitation of the impaired tissues, by reason of the action of a local irritant; the deduction is logical, therefore, that additionally increased hyperaction of the parts, by inducing a corresponding exaggeration of functional activity that cannot be healthfully sustained, must produce, in a corresponding ratio, a more rapid disintegration of the implicated tissue. The correctness of this view is attested by the history of this case, and is confirmed by experience in all cases where the local malady is an issue of impaired innervation, or greatly diminished textural tonicity.

We repeat, then, that the curative measures upon which the result will mainly hinge must be those addressed to the failing powers of the system—to a re-establishment of textural tonicity through the functions of nutrition—to a correction of the secretions—to increased innervation, and by whatever means the parts involved may be improved in their defences against the further encroachments of the disease. The remedies chiefly relied upon, in the present case, were quinine, brandy and cod-liver oil, variously combined, to meet particular indications, with also such nutritious articles of diet as were best suited to the case. These remedies were given as freely as the child would bear them, and persisted in without faltering. To them, we believe, not without good reason, the little patient is indebted for a prepossessing face, without blemish or mutilation, and perhaps for her life.

After a gradual improvement in the general condition of the patient was obtained, the recovery of the parts were aided by such local applications as best promoted a healthy action in the parts, by which their renewal was accelerated, whilst the mouth was kept free as possible from offensive accumulations.

This child, since her recovery, has had frequent attacks of her former complaint, which have been promptly arrested by the family physician. There has been no tendency to a renewal of the local difficulty.—*Dental Register.*

